# The functions of self‐harm in young people and their perspectives about future general practitioner‐led care: A qualitative study

**DOI:** 10.1111/hex.13733

**Published:** 2023-02-16

**Authors:** Faraz Mughal, Carolyn A. Chew‐Graham, Opeyemi O. Babatunde, Benjamin Saunders, Aula Meki, Lisa Dikomitis

**Affiliations:** ^1^ School of Medicine Keele University Keele UK; ^2^ Division of Psychology and Mental Health, Centre for Mental Health and Safety University of Manchester Manchester UK; ^3^ Specialist Psychotherapies Service Birmingham and Solihull Mental Health NHS Foundation Trust Birmingham UK; ^4^ Kent and Medway Medical School University of Kent and Canterbury Christ Church University Canterbury UK

**Keywords:** general practitioners, interviews, nonsuicidal self‐injury, self‐injurious behaviour, suicide attempt, young adult

## Abstract

**Background:**

Self‐harm in young people is a serious concern but a deeper understanding of the functions of self‐harm in young people can tailor care and inform new clinical interventions to reduce repeat self‐harm and suicide risk. General practitioners (GPs), as frontline healthcare professionals, have an important role in managing self‐harm in young people. This study aimed to explore the functions of self‐harm in young people and their perspectives on future GP‐led care.

**Methods:**

A qualitative study using interviews with young people aged between 16 and 25 years with a personal history of self‐harm was conducted. Interviews were transcribed and analysed using reflexive thematic analysis.

**Findings:**

Four distinct functions were identified: (1) handling emotional states; (2) self‐punishment; (3) coping with mental illness and trauma; and (4) positive thoughts and protection. Young people valued GP‐led support and felt future GP interventions should include self‐help and be personalised.

**Conclusions:**

These findings support clinicians, including GPs, to explore the functions of self‐harm in young people aged 16–25 in a personalised approach to self‐harm care. It should be noted that self‐harm may serve more than one function for a young person and thus interventions should recognise this.

**Patient and Public Contribution:**

A group consisting of young people with lived experience of self‐harm, carers, the public, and those who work with young people who harm themselves conceived this study idea, informed recruitment methods and the interview topic guide, and supported the interpretation of findings.

## 1 INTRODUCTION

Self‐harm is defined as self‐injury or self‐poisoning irrespective of suicidal intent, and in young people, self‐harm is a growing concern of international importance.^1^, ^2^ Self‐harm in young people is associated with repeat self‐harm, anxiety, and depression,^3^ and self‐harm is the strongest risk factor for death by suicide.^4^ In young people suicide is a leading cause of death.^5^ Around one in four young people, aged 10–24 years in England, have previously harmed themselves,^6^ and in young people who die by suicide, over three quarters had a history of self‐harm.^7^

In the United Kingdom (UK) one in five young people who self‐harm in general practice, repeat self‐harm the following year.^8^ Risk factors for self‐harm in young people include bullying, family discord, and mental health difficulties.^2^, ^9^ In young people, cutting is the most common method of self‐harm with medication overdose next, but many young people report multiple methods of self‐harm.^10^, ^11^ Young people in the UK have described their self‐harm to have started 10 years before the recent repeat self‐harm.^10^, ^12^

In 1989, Favazza described why some patients harmed themselves and this included control of sexual desires, establishing control, and relief from alienation.^13^ The functions of self‐harm have been discussed in substantial reviews.^14^, ^15^ Nock proposed a four‐functional model informed by behavioural psychology: intrapersonal negative reinforcement (reduction in unhelpful thoughts), intrapersonal positive reinforcement (increase in satisfying thoughts), interpersonal positive reinforcement (increase in social attention), and interpersonal negative reinforcement (reduction in undesired social demands).^15^ In a meta‐analysis, intrapersonal functions of self‐harm were found to be more common than interpersonal functions, in young people aged 9–14 years.^16^ In a systematic review, Edmondson et al. presented a descriptive framework incorporating past literature (Suyemoto and Nock) and identified 15 functions of non‐suicidal self‐harm in people aged 10–92 years across three broad themes: responding to distress, self‐harm as a positive experience, and defining the self.^17^

In a meta‐synthesis of experiences of self‐harm in 12–18‐year‐old adolescents, four themes were identified: self‐harm to obtain release, controlling challenging feelings, representing unacceptable feelings, and connecting with others.^18^ When considering the effect of interpersonal stressors on young people's self‐harm, worries about family breakdown, parental responses to self‐harm, and bullying from peers, were all described as contributing to self‐harm behaviour in young people.^10^

Reasons for self‐harm in young people can be multiple, complex, and often contradictory, with explanations and motivations for self‐harm changing with time, place, and social context.^12^, ^17^ It is challenging for young people to convey, in turn, this complexity and explain why they self‐harm, and for clinicians and health professionals to understand self‐harm, and therefore tailor interventions to young people.^19^ The National Institute for Health and Care Excellence (NICE) self‐harm clinical guideline recommends research exploring what self‐harm means to people.^1^ Reducing rates of self‐harm in young people is an international priority.^20^ Functions of self‐harm have been described—as highlighted above—in adolescents and adults but have yet to be explored in young people aged 16–25 years explicitly. A deeper understanding about the functions—what self‐harm means and its purpose—of self‐harm in young people 16–25 years through qualitative methods can lead to nuanced new meanings of self‐harm in these young people to tailor care and inform the development of clinical interventions, to reduce further self‐harm and improve the quality of life in this vulnerable group of patients.^21^, ^22^ This would also address the above‐mentioned NICE research recommendation.

Young people, aged 16–25, often first seek help from family physicians and general practitioners (GPs) with a history of self‐harm and these clinicians can identify and intervene early to prevent repeat self‐harm in young people.^23^ GPs/family physicians have an important role in the management of self‐harm in young people and they can deliver holistic care, focusing on the function self‐harm has, for individual young people.^23^, ^24^ GPs are however limited in what they can offer to young people who have self‐harmed in the consultation, especially in the context of a lack of accessible effective interventions which is a missed opportunity for early intervention.^25^, ^26^ It is thus important to seek and understand the views of young people about future GP‐led support and what they feel may help for their self‐harm. Understanding the views of young people can lead to improvements in GP‐led care and in developing acceptable GP‐led interventions, targeting the function of self‐harm, for young people that are more likely to work in the real world.

Our research questions were: what are the functions of self‐harm for young people aged 16‐25 and what are their thoughts about future GP delivered care for self‐harm in the consultation. The aims of the study were therefore to explore:

1. the functions of self‐harm in young people aged 16–25 years
2. young people's perspectives about future GP‐led support for self‐harm.

## 2 METHODS

This qualitative study was conducted from April to November 2019, and we used in‐depth semi‐structured interviews to explore young people's reasons for self‐harm and thoughts about GP‐led support for self‐harm. This study is reported according to the Standards for Reporting Qualitative Research.^27^

### 2.1 Participant recruitment

We adopted an inclusive approach to self‐harm research, encouraged by the study's patient and public involvement (PPI) advisory group, where all types of self‐harm (e.g., self‐poisoning, cutting, burning, pinching) were included, irrespective of suicidal intent. Young people would be eligible to participate if they were 16–25 years old, had harmed themselves previously, lived in England, and were able to provide informed consent and be interviewed in English.

A diverse sample of young people from England was targeted through social media (Twitter), community recruitment, and national self‐harm third‐sector organisations. Tweets were drafted and refined with input from the study's PPI group and were shared on the lead author's (F. M.) personal Twitter account to generate participant interest in the study. The study recruitment poster was displayed on university campuses and college settings and in libraries.

A list of self‐harm third‐sector services and groups in England was compiled from an internet search and knowledge of PPI group members. Organisations were contacted by email with a description of the study and asked if they would be willing to share the recruitment poster within their services. Table 1 lists the organisations contacted and their geographic location.

**Table 1:** Third‐sector self‐harm organisations contacted.

Third‐sector organisations	Geographical location (UK)
Harmless	Nottingham
ECHO	Stoke‐on‐Trent
Battle Scars	Leeds
The Lowdown	Northampton
Self‐Harm Awareness For All	Cumbria
42nd Street	Manchester
Your Emotional Support Services	Staffordshire
Shropshire Mind	Shrewsbury

Interested eligible individuals made contact by email or on Twitter; and FM subsequently emailed a participant information pack (invitation letter, consent form, and research information sheet) and answered individual questions about the study. FM arranged the location, format, and time of interviews with individuals who wanted to participate.

### 2.2 Data collection

In‐depth semi‐structured interviews with participants were conducted; and allowed for the exploration of participants' perspectives, guided by an interview topic guide (see Table 2), while remaining flexible to participants' accounts enabling exploration of unexpected areas.^28^ The interview topic guide was informed by the literature, input from members of the PPI group, and the research team.

**Table 2:** Interview topics discussed with participants.

Interview topics
1. Ask young person to say a little about themselves
2. Ask about self‐harm: how long, why, and what self‐harm means to self
3. What makes young person seek help for self‐harm
4. Thoughts on GP led support and care in the consultation and what may help

Potential participants were given the option of a telephone or face‐to‐face interview. Face‐to‐face interviews were conducted in private university meeting rooms. At the start of the interviews participants received a ‘Staying Safe Sheet’ which listed support services for self‐harm. All interviews began with the researcher asking the young person how long they have been harming themselves, and this allowed participants to reply in as much detail as they wished, supporting rapport building.^29^

All interviews were conducted by the lead researcher (F. M.) who declared his professional identity: a GP with clinical and research expertise in self‐harm in young people, to all participants. Interviews were digitally recorded and transcribed verbatim by F. M. or a professional company. Transcripts were pseudonymised with consent. Data saturation (the degree to which new data repeats what was previously expressed in past data at the transcript level) was achieved after 13 interviews,^30^ and data collection therefore stopped.

### 2.3 Data analysis

We analysed interview transcripts through reflexive thematic analysis.^31^ Reflexive thematic analysis was guided by the six stages of thematic analysis by Braun and Clarke,^31^ and placed researchers' subjectivity central to knowledge production, requiring a more active, flexible and collaborative approach to coding, and generating themes within a fluid and recursive analytical process.

FM led the analysis and coding of all transcripts, reading and rereading transcripts for familiarity. Each transcript was independently coded twice across research team members. Recurring codes across transcripts informed wider categories within an analysis framework that included relevant data extracts on which all research team members commented and helped to refine. Higher level recurring themes were then agreed upon within the research team. Analysis was undertaken in Microsoft Word and QSR NVivo 12.^32^ The researchers reflected, during study meetings, on how both their disciplinary and personal backgrounds influenced their assumptions and perspectives about self‐harm, and on the interpretation of data and findings.

### 2.4 PPI

A specific self‐harm in young people's PPI study advisory group was formed and led by the lead researcher (F. M. at Keele University) to inform all study stages. The PPI study group comprised of eight members and consisted of young people (16–25 years) with lived experience of self‐harm, parents and carers of young people who self‐harm, third‐sector self‐harm support workers, and public members. The group met in person and remotely five times over 3 years, with meetings chaired by F. M. and co‐facilitated by a Keele University PPI support officer.

At the first PPI meeting, members recognised that self‐harm may have different functions for young people, and this contributed to the conception of this study. Members of the group supported the design of the research information sheet and interview topic guide, informed recruitment strategies, and supported the interpretation of findings. PPI group members were reimbursed for their time and travel in accordance with National Institute for Health and Care Research guidance.^33^

## 3 FINDINGS

Thirteen young people with self‐harm behaviour were interviewed: interviews ranged from 25 to 49 min (mean: 36 min). The age of participants ranged from 19 to 25 (mean: 22 years): 12 young people identified as female and one as transgender male. Participants were from the West Midlands, East Midlands, and South East of England. Seven young people were White British, three mixed ethnicity, one Asian British, one White American, and one young person chose not to disclose ethnicity. There was no identified distress during the recruitment or data collection process, and the study risk protocol was not activated.

Young people provided their first ages of self‐harm which ranged from 8 to 23 years. Cutting was the most common (92%) method of self‐harm in participants and more than half (54%) of participants had experience with more than one method of self‐harm. Self‐poisoning was mentioned by three participants. Participant demographic characteristics, disclosed ages of first self‐harm, and types of self‐harm are stated in Table 3.

**Table 3:** Demographic characteristics, ages of first and types of self‐harm.

Participant ID	Gender	Age	Disclosed age of first self‐harm	Types of self‐harm
1	F	24	23	Cutting; medication overdose
2	F	24	9/10	Cutting
3	F	21	13	Cutting; burning
4	F	23	12/13	Cutting; burning; hair pulling; scratching
5	F	22	16	Cutting
6	F	23	11/12	Cutting; scratching
7	F	25	13	Cutting; burning; scratching; medication overdose
8	F	25	11	Scratching
9	M	21	17	Cutting
10	F	19	11	Cutting
11	F	22	18	Cutting
12	F	19	14/15	Cutting; medication overdose; scratching; hitting self
13	F	19	eight	Cutting; burning; hair pulling; scratching

Abbreviations: F, female; ID, identifier; M, male.

We generated four higher‐level themes representing the functions of self‐harm in young people: handling emotional states, self‐punishment, coping with mental illness and trauma, and positive thoughts and protection. These functions are listed and described in Table 4. One further theme was generated: valuing GP support and thoughts on GP interventions. Overall, five themes are presented below, each with illustrative participant quotations identified by a participant identifier number.

**Table 4:** Summarising the functions of self‐harm in young people aged 16–25.

Handling emotional states	Self‐punishment	Coping with mental illness and trauma	Positive thoughts and protection
Recognising emotions after and between self‐harm acts	Justifying internal pain and distress	As a means of handling active mental illness	Feeling in control when struggling with emotions
Expressing overwhelming emotional distress	Not meeting personal expectations	Coping with past trauma	Therapeutic nature of self‐care after self‐harm
Converting emotional distress into a physical stimulus	Being critical of self		As a means to protect self from death by suicide
Avoiding and suppressing distress			

### 3.1 Handling emotional states

Young people described self‐harm to be a method of actively managing emotional states, predominantly distress, and consisting of recognising emotions, expressing them, converting emotional pain into physical pain, and avoiding and suppressing difficult thoughts.

Some young people felt self‐harm enabled them to recognise their emotional state when they self‐harmed, and reflected on states changing between episodes of repeat self‐harm:

‘… it basically is the result of years and years of low mood, depression, suicidal ideation…’ (P1, 24 years).
‘I classify my self‐harm in two different ways, so I have like I have like a manic kind of “I'm feeling too much” like I'll slash a lot so it will be lots of stuff, lots of self‐harm, or it will be “I am feeling very numb and absent”…’ (P3, 21 years).

For other young people self‐harm allowed them to communicate emotional distress that was overwhelming to themselves, and to others:

‘…the main reason obviously was I felt like I was alone, sort of, didn't have any other way of getting the feelings out’ (P10, 19 years).

Self‐harm by cutting for some young people allowed the conversion of emotional pain and distress into a physical stimulus which, in turn, can ‘ground’ young people:

‘I needed to really ground myself all of a sudden, just to feel in the moment…’ (P11, 11 years).
‘I would dissociate a lot, I would just be completely out of it, and it would kind of wake me up because it was some like extreme stimulus, so it would wake me up’ (P12, 19 years).

For some participants self‐harm served a function of avoiding emotional pain and distress by distraction from it: ‘like it's a distraction, like away from all my thoughts…’ (P5, 22 years); and by suppressing distressing thoughts: ‘so it was very much sort of keeping me… sort of numb the pain, make these feelings that were so overwhelming sort of go’ (P9, 21 years).

The above data extracts highlight that managing emotions serves as a key purpose for young people's self‐harm, but often emotions experienced differ for different young people. This variation in experiences among young people needs to be taken into account when consulting young people who present with self‐harm to clinical services.

### 3.2 Self‐punishment

Young people reflected on an important function of self‐harm being self‐punishment, to justify internal emotional pain, and because of failure to meet individual expectations, and self‐criticism:

‘It's because I feel like I need to punish myself, like I feel like I'm not, what's the words, because I'm not outwardly suffering in a sense, it's all internally, I feel like I have to physically manage this in order to justify it for myself’ (P6, 23 years).
‘…I just think it was a way of projecting like distress onto myself and like taking it’ (P13, 19 years).

Some young people described that they were self‐critical, and this resulted in self‐harm:

‘It was a lot about the sort of the inability to be everything I expected of myself…’ (P4, 23 years).
‘Ermm, sometimes a bit of self‐punishment cos I'm my own worst critic’' (P11, 22 years).

Participants described not meeting personal expectations and being self‐critical as drivers for their self‐harm. In this fast changing, technology orientated world young people are living in, there are likely societal pressures influencing personal targets which are important to acknowledge. Young people can also be reminded about alternative healthier ways to alleviate internal distress.

### 3.3 Coping with mental illness and trauma

Some participants described how self‐harm helped them cope with mental illness and trauma:

‘So I had some elements of PTSD… the things that cause the PTSD are the biggest motivators for me to engage in self‐harming behaviour. Ermm I think when I'm feeling really low or numb or just upset over myself that is often what I go to’ (P2, 24 years).
‘When I was younger, I got bullied horrendously… I was a big, fat little ball erm so I got bullied a lot because I went to a private school… erm so I got bullied from a very young age’' (P13, 19 years).

The identification of self‐harm being undertaken to cope with coexisting mental illness and past trauma is important but concerning. It highlights that self‐harm is being used as a coping strategy which can lead to detrimental complications, including suicide, for young people. It magnifies the importance of treating coexisting mental illness and trauma alongside self‐harm behaviour in primary care and mental health services.

One young person shared that self‐harm served a self‐medicating function when they were dealing with ongoing mental health difficulties such as anxiety:

‘When I'm very down and isolated and on my own, when thoughts come, and “cause I struggle with anxiety a lot as well…. but yeah that's when it [self‐harm] normally comes about when I have hit that really down”’ (P5, 22 years).

### 3.4 Positive thoughts and protection

Young people stated that a key function of self‐harm was to feel in control amid the difficulty of changing and overwhelming emotions:

‘It means I have my control back, suddenly I'm not being pulled in one direction and another by my emotions’ (P11, 22 years).
‘Erm it was something that was mine, I know that sounds very strange but erm like it wasn't something someone could take away from me… so I guess controlling I guess a control mechanism kind of thing’ (P13, 19 years).

There was a therapeutic function to self‐harm:

‘But there's also an element of self‐care afterwards cos the tidying of the wounds, it's kind of therapeutic just to feel that I'm caring for myself’ (P11, 22 years).

Participants felt self‐harm protected them from suicide by helping them deal with suicidal thoughts:

‘and it very much became a part of my life and it seemed like no one kind of understood why I done it and to me it was a very positive thing at the time and it was stopping me from taking my own life’ (P9, 21 years).
‘for me stopping was not an attractive solution as it was helping me cope with other things like suicide and… thoughts and… stuff’ (P1, 24 years).

### 3.5 Valuing GP support and thoughts on GP interventions

In response to being asked about GP‐led interventions, young people shared that they would value GP support and intervention, immediately in the consultation, and regularly while being placed on waiting lists for specialist support. They recognised that when seeing a GP, it is ‘a good space… and good time to be offering an intervention’ (P7, 25 years).

Some young people reflected on how useful GP support and intervention would be while they wait for further support or specialised help outside of general practice:

‘That is a really good idea actually erm, the whole waiting cause like there's a waiting list, like every time I've been somewhere to get help, there's been a waiting list’' (P5, 22 years).

Young people stated that GP‐led interventions for self‐harm should enable GPs to establish and build rapport with them which can result in the young person not feeling alone:

‘…I think if there was some sort of intervention or some sort of tool or app or, or website or whatever but like it's about trying to build a rapport with young people’ (P4, 23 years).
‘Also, just them knowing that there are multiple options, multiple different things that can help them and that, yeah the fact that GPs are like this prepared for it means that they're not alone’ (P10, 19 years).
‘…that would be really good because then you can go back and get the check up and everything but it would also be good for the patients as well because like they'd be able to understand where they are…’ (P5, 22 years).

Some participants suggested that future GP interventions for self‐harm in young people should include offering young people distraction techniques:

‘Cos I had to wait until I'd met a counsellor who you know, helped me develop coping mechanisms and recommended ones that might work. You know, GPs generally didn't have any idea about that sort of thing, and it can be as simple as… a walk every evening’ (P11, 22 years).
‘I would have definitely welcomed it, because I didn't know anything about it, everyone was telling me saying need to replace with healthy coping strategies, I didn't know what healthy coping strategies were…I would have appreciated…just one single advice’ (P1, 24 years).

Young people also stated that GP interventions should include providing self‐help; and care should be personalised to the young person by allowing young people the opportunity to talk:

‘That would be really, really useful erm, actually maybe understanding of what a young person's self‐harm actually is and what things I could do to, other things I could do apart from that… if information is actually given to the younger person, than actually being left in the dark’ (P9, 21 years).
‘It would be more specialised, and it would be more direct and like allocated and accurate for someone’ (P3, 19 years).

## 4 DISCUSSION

### 4.1 Summary of findings

Four distinct functions of self‐harm were identified in this group of young people aged 16‐25 years: handling emotional states, self‐punishment, coping with mental illness and trauma, and positive thoughts and protection. Self‐harm for some young people enabled them to manage emotional distress: to recognise it, express it, convert it into physical pain, and to suppress overwhelming emotional suffering. In young people self‐punishment is a function of self‐harm and can be because of self‐criticism. Self‐harm can enable young people to cope with mental illness and trauma. A key function of self‐harm for young people is the feeling of being in control and protecting themselves from suicide. Young people described how GP‐led interventions can be helpful and should build rapport between the GP and young person, offer self‐help, and be personalised.

### 4.2 Comparison with existing literature

Miller et al. reported that self‐harm in 13–17‐year‐old females regulated emotions and held a protective function which is similar to two of the functions found in our study of slightly older young people; but they also described an addictive element to self‐harm in young adolescents which we did not discover.^34^ The four functions we identified would be classified according to Nock, as intrapersonal functions as opposed to interpersonal functions, suggesting that relationship triggers don't play a substantial role for these young people.^15^ In people with an age range of 19–57 years and history of repeat self‐harm four functions were identified: managing my mental state; communicating distress; distracting from suicidal thoughts; and producing positive feelings.^35^ These functions align with the two functions we identified in young people aged 16–25 years: handling emotional states and positive thoughts and protection. This may insinuate that the functions for self‐harm in 16–25 year old young people, particularly around self‐harm as a means of managing emotional distress and protecting from suicide, persist into adulthood and this highlights the importance of identifying and supporting people who self‐harm with their distress and suicidal thoughts as early in their life as possible to prevent ongoing self‐harm behaviour. In the context of Favazza's early finding (1989) that self‐harm can be a means of regaining control for an individual, this purpose has appeared to have continued over time and remains a means for some 16–25 year olds, but we found no evidence of self‐harm as a means of controlling sexual urges.^13^

Young people have previously suggested that self‐help materials from GPs would be helpful and this aligns to our findings, however concerns about time in the consultation were noted.^36^ GPs have highlighted the importance of communication and relationship building with young people at risk of self‐harm and it's important to know that young people in this study felt future GP‐led treatments should enable the building of rapport which may help with feelings of social connectedness.^37^ In an Australian focus group study of young people about care for suicidal behaviour/self‐harm in primary care, participants described wanting to be shown how to use tangible resources in the consultation with a GP, but not all participants had personal experience of self‐harm.^24^ While practical resources are likely helpful for young people who have self‐harmed, GPs and family physicians should personalise them and offer distraction techniques to help the young person handle urges to self‐harm.

### 4.3 Strengths and limitations

While functions for self‐harm have been explored in adolescents and adults, to our knowledge this is the first study to identify functions of self‐harm in young people aged between 16 and 25 years specifically. This study was originally conceived through discussion with members of the PPI group; and they informed study design, participant facing documents, and recruitment methods, thereby improving the relevance and credibility of our findings. Team members have professional backgrounds in sociology, medical anthropology, clinical psychology, evidence synthesis, applied health research, and general practice, and conducting analysis with researchers from different professional backgrounds enhances the trustworthiness of our findings.^38^

There are, however, some limitations. The participants were all aged 19 years or older and so the voices of 16–18‐year‐old young people are missing: this is an important group of young people who are most often in full‐time education and are navigating entering university or employment. Participants were mostly female and thus future research which explores the perspectives of the functions of self‐harm in male young people, including those from ethnic minority backgrounds, is crucial. The interviewer declared his professional background before participant interviews: talking about one's background can support the trust and rapport building process when gathering interview data, however, it may have influenced the content of participants' accounts, such as when reflecting on GP‐led care.^39^ We asked participants about reasons for self‐harm in the context of all their self‐harm episodes, recognising motivations for self‐harm often change; we did not seek to understand motives for individual self‐harm acts, which could include different methods, and in turn, were unable to link motives to the chronology of self‐harm methods and episodes.

### 4.4 Implications for practice and research

These identified functions in young people can help GPs and healthcare professionals further their understanding about self‐harm. They can support conversations with young people because exploring and uncovering the function(s) of self‐harm in a young person should tailor the care subsequently offered. GPs and primary care clinicians should take time in practice to ascertain the function(s) of self‐harm for the young people they consult: for example, if the function is predominantly around managing emotional states, then the clinician may guide the young person to emotion regulation techniques; or if the function of self‐harm is protective from suicidal thoughts, the GP can work with the young person on alternative ways to handle suicidal thoughts.

At present, promising interventions for reducing self‐harm episodes in young people include dialectical behaviour and mentalisation‐based therapies which focus on one treatment lens, for example regulating emotions. We identified functions that can provide an alternative way to intervene for young people where the focus may not entirely be on affect regulation but tailored to the specific function and thus needs to be identified (such as concurrent mental illness or past trauma) and therefore personalised to the young person. For some young people, self‐harm served more than one function, and this is important to acknowledge in the adaptation and delivery of interventions. GPs need to consider building rapport and tailor self‐harm care to the function(s) self‐harm serves for each young person in a person‐centred approach. This will be challenging amid the current pressures on primary care but this vulnerable group of young people deserve better care.

A functional analysis of self‐harm in young people should inform the development of future clinical interventions for self‐harm. It is important that research on the functions of self‐harm in male young people is undertaken, and a qualitative approach would allow for rich insights to be captured. Exploring functions in young people who self‐harm multiple times would be valuable and would inform the development of clinical interventions for young people with repeated self‐harm behaviour specifically. In the development of future GP‐led interventions the views of young people should be incorporated, as well as GPs, to increase the likelihood of interventions being meaningful and acceptable to young people within a primary care context.

## AUTHOR CONTRIBUTIONS

This study idea was conceived by Faraz Mughal, Carolyn A. Chew‐Graham, Opeyemi O. Babatunde, and Lisa Dikomitis. Faraz Mughal led the data collection and analysis. All authors contributed to data analysis and interpretation of findings. Faraz Mughal drafted a first version of the manuscript. All authors critically edited the manuscript and agreed on the final version for submission.

## CONFLICT OF INTEREST STATEMENT

The authors declare no conflict of interest. Faraz Mughal was a member of the 2022 NICE self‐harm clinical guideline development committee. Lisa Dikomitis was awarded a Senior Fellowship by the Higher Education Academy. Carolyn A Chew‐Graham is part‐funded by the West Midlands NIHR Applied Research Collaboration. The views expressed in this article are those of the authors and not necessarily those of the NHS, NICE, NIHR, or the Department of Health and Social Care.

## ETHICS

This study received ethical approval from Keele University's Ethical Review Panel (REF2417). All participants provided written informed consent, which was an ongoing process, and were free to withdraw from the study up to 1 week after the interview. Participants were offered an online shopping voucher as a token of appreciation for their participation in the study.

## Data Availability

Data are available upon reasonable request to the authors.
